# Simultaneously inactivating Src and AKT by saracatinib/capivasertib co-delivery nanoparticles to improve the efficacy of anti-Src therapy in head and neck squamous cell carcinoma

**DOI:** 10.1186/s13045-019-0827-1

**Published:** 2019-12-05

**Authors:** Liwei Lang, Chloe Shay, Xiangdong Zhao, Yuanping Xiong, Xuli Wang, Yong Teng

**Affiliations:** 1 Department of Oral Biology and Diagnostic Sciences, Dental College of Georgia, Augusta University, 1120 15th Street, Augusta, GA 30912 USA; 2 Department of Pediatrics, Emory Children’s Center, Emory University, Atlanta, GA USA; 3 Department of Radiology and Imaging Sciences, School of Medicine, University of Utah, Salt Lake City, UT USA; 4 Georgia Cancer Center, Department of Biochemistry and Molecular Biology, Medical College of Georgia, Augusta University, Augusta, GA USA; 5 Department of Medical Laboratory, Imaging and Radiologic Sciences, College of Allied Health, Augusta University, Augusta, GA USA

**Keywords:** HNSCC, Src, AKT, Saracatinib, Capivasertib, Co-delivery nanoparticles, Synergistic effects

## Abstract

**Background:**

Src, an oncoprotein that drives progression of head and neck squamous cell carcinoma (HNSCC), is commonly hyperactivated in this disease. Unfortunately, the clinical benefit of targeting Src is significantly dampened in HNSCC patients, because the cytotoxic effects of anti-Src therapy and tumor resistance to it are less predictable. Thus, understanding the mechanism of tumor resistance to Src inhibition and seeking a way to overcome it are warranted.

**Methods:**

Dual drug-loaded nanoparticles (NPs) were developed to co-deliver Src inhibitor saracatinib (AZD0530) and AKT inhibitor capivasertib (AZD5363) into the same population of tumor cells. An orthotopic tongue tumor model was generated to evaluate the in vivo therapeutic effects. Cell growth was determined by CellTiter-Glo® Luminescent Cell Viability Kit, colony formation, and 3D culture, and tumor growth was determined by bioluminescence and tumor size. The molecular changes induced by the treatments were assessed by Western blotting and immunohistochemistry.

**Results:**

Capivasertib inactivated the AKT-S6 signaling and re-sensitized saracatinib-resistant HNSCC cells to saracatinib. Combination of capivasertib with saracatinib suppressed HNSCC growth more efficiently than either drug alone. Cathepsin B-sensitive NPs for co-delivering saracatinib and capivasertib significantly improved the efficacy of tumor repression without increasing side effects, which were due to highly specific tumor-targeting drug delivery system and synergistic anticancer effects by co-inactivation of AKT and Src in HNSCC cells.

**Conclusions:**

Addition of AKT blockade improves anti-HNSCC efficacy of anti-Src therapy, and co-delivery of capivasertib and saracatinib by tumor-targeting NPs has the potential to achieve better treatment outcomes than the free drug combination.

## Background

Head and neck squamous cell carcinoma (HNSCC), a malignancy arising in the mucosal lining of the oral cavity, pharynx, and larynx, ranks as the sixth most common cancer worldwide. Aggressive treatment for HNSCC can induce severe adverse outcomes (e.g., mastication dysfunction, altered speech, and facial disfigurement), and to date, the long-term survival of HNSCC patients remains poor [1–3]. As little progress has been made, new anti-HNSCC approaches are required to improve overall clinical outcomes.

Many biologic compounds have been developed to target specific cellular domains of cancer cells. The targets include Src, a non-receptor tyrosine kinase that acts as a signal transducer from cell surface receptors by sequential phosphorylation of tyrosine residues on substrates [4, 5]. Src is overexpressed and highly activated in HNSCC and is strongly associated with solid tumor progression, metastasis, and poor outcomes [6, 7]. Anti-Src agents dasatinib and saracatinib (AZD0530) are currently in clinical development for patients with solid tumors. However, these Src inhibitors exhibit limited activities against HNSCC in clinical trials despite consistent Src inhibition [8–10]. These treatment failures may be attributed to cancer heterogeneity and molecular complexity, implying that the use of a single therapeutic agent is not sufficient to halt cancer progression. Thus, there is a need for studies to develop combined therapy that overcomes the limitations of monotherapy.

Saracatinib and dasatinib are both tyrosine kinase inhibitors selective for Src through reversible ATP competition for the enzyme domain, which leads to the impairment in Src tyrosine phosphorylation [5, 11]. We showed previously that saracatinib inhibits Src activation in HNSCC cells more efficiently than dasatinib [5]. In the present study, we examined the mechanism responsible for saracatinib activity and develop a promising therapeutic approach to inhibit saracatinib-resistant (Sar-R) HNSCC. We show that saracatinib incompletely inactivates AKT, which is one of the leading causes of HNSCC cell resistance to Src-targeted treatment. Addition of AKT inhibitor capivasertib (AZD5363) to sarcatinib can simultaneously inactivate the AKT-S6 axis and Src signaling in HNSCC cells, leading to enhanced treatment outcomes than monotherapy. Moreover, we developed tumor-targeting nanoparticles (NPs) to co-deliver capivasertib and saracatinib into the same population of HNSCC cells in a preclinical mouse model, which significantly improved the efficacy of HNSCC repression without systemic toxicity. The new combination regimen explored in this study may better exert anti-HNSCC activity in clinical settings with the significantly combined influence of pronounced tumor-targeting properties and synergistic cytotoxic effect of Src and AKT co-inhibition.

## Methods

### Cell lines and cell culture

DU145, PC3, MDA-MB-231, T47D, H1299, and H1661 cells were directly purchased from ATCC and were authenticated by Human STR Profiling Cell Authentication Service. HN6, HN8, HN12, HN13, and HN17 cells were a gift from Dr. W. Andrew Yeudall [12, 13]. All cells were used for experiments before passage 10 and cultured in DMEM medium containing 10% fetal bovine serum at 37 °C in a humidified incubator supplied with 5% CO2.

### Antibodies, molecular reagents and standard assays

Antibodies that recognize phosphorylated or total proteins, including Src, p-Src (Y416), AKT, p-AKT (S473), S6, p-S6 (S240/244), ERK1/2, p-ERK1/2 (T202/Y204), FAK, p-FAK (T397), EGFR, p-EGFR (T1068), STAT3, STAT3 (T705) were purchased from Cell Signaling Technology (Beverly, MA). β-actin antibody and D-Luciferin bioluminescent substrate were purchased from Sigma-Aldrich. Saracatinib and capivasertib were purchased from Selleckchem (Houston, TX). Plasmid HA-AKT-DN (K179 M) (Addgene, Plasmid #16243) and HA-AKT-CA (Addgene, Plasmid #16244) were a gift from Dr. Mien-Chie Hung (Addgene, Plasmid #16243) [14]. Standard cell culture, plasmid transfection, Western blotting, colony formation, CellTiter-Glo® Luminescent Cell Viability and CellTiter 96® AQueous One Solution Cell Proliferation Assay (MTS) assays were carried out as previously described [2, 3, 5].

### Development of luciferase stable HNSCC cells

Luciferase stable HN8 and HN12 cells (HN8-luc and HN12-luc cells) were generated by transduction of pGL4.5 vector (Promoga, Madison, WI) and selection of 150 μg/ml hygromycin (Sigma-Aldrich, St Louis, MO) for 4-5 weeks. Firefly and *Renilla* luciferase activity was measured by the Dual-Glo^TM^ Assay System (Promega).

### Three-dimensional (3D) cell cultures

Briefly, 1 × 10^5^ HNSCC cells were seeded into SeedEZ^TM^ scaffold (Lena Bioscience, Atlanta, GA) pre-coated with Poly-D-Lysine solution (Sigma-Aldrich, St. Louis, MO) [15]. After 10 days of culture, 3D cultures were incubated with free or NP-associated drugs for 12 days. At the endpoint of this experiment, cells in SeedEZ^TM^ scaffold supplied with complete medium were stained with Texas Red®-X phalloidin (Invitrogen), followed by fluorescence imaging (Zeiss). Cell viability in 3D cultures was quantified by an alamarBlue assay (Bio-Rad).

### Generation of Sar-R HN8 cells

Briefly, HN8 cells were treated with IC90 dose of saracatinib (20 μM) and maintained in the medium containing IC50 dose of 2 μM for 5 generations. The dose was gradually increased by 1 μM every 2 or 3 weeks until the maximum tolerated dose of 5 μM was reached.

### Solid-Phase Peptide Synthesis

Synthesis of the peptide was carried out using the Fmoc strategy manually in a glass reaction vessel fitted with a sintered glass frit using 2-chlorotritylchloride. Coupling reactions were performed manually by using 2 equiv of N-Fmoc-protected amino acid (relative to the resin loading) activated in situ with 2 equiv of PyBOP and 4 equiv of diisopropylethylamine (DIPEA) in DMF (10 mL/g resin). The coupling efficiency was assessed by the Kaiser test. N-Fmoc protecting groups were removed by treatment with a piperidine/DMF solution (1:4) for 10 min (10 mL/g resin). The process was repeated three times and the completeness of deprotection verified by UV absorption of the piperidine washings at 301 nm. Synthetic linear peptides were recovered directly upon acid cleavage. Before cleavage, the resin was washed thoroughly with methylene chloride. The linear peptides were then released from the resin by treatments with a solution of acetic acid/trifluoroethanol/methylene chloride (1:1:8, 10 mL/mg resin, 2 × 30 min). Hexane (5-10 volumes) was added to the collected filtrates, and the crude peptides were isolated after concentration as white solids. The residue was dissolved in the minimum of methylene chloride and diethyl ether was added to precipitate peptides, followed by triturated and washed three times with diethyl ether to obtain crude materials. Peptide was further purified by preparative HPLC prior to conjugation.

### Development and characterization of the saracatinib/capivasertib co-delivery NPs

Linear-dendritic mPEG5000-BMA4 containing four branches of amine groups, the cathepsin B (CTSB)-sensitive polymeric drug carrier, was synthesized as described previously [5]. To prepare single drug-loaded NPs, hydrophobic drugs (saracatinib or capivasertib) were loaded into NPs by the solvent evaporation method. Briefly, drug (1.0 mg) and amphiphilic polymer (10 mg) were first dissolved in anhydrous chloroform/methanol (1/1) in a 10 mL round bottom flask. The solvent mixture was evaporated under vacuum to form a thin film. PBS buffer (1 mL) was added to re-hydrate the thin film, followed by 30 min of sonication. Free drugs not associated with the NPs were removed by running the NP solutions through centrifugal filter devices (MWCO: 3.5 kDa, Microcon®). The drug-loaded formulation on the filters were recovered with PBS. To prepare co-delivery NPs (NP-com), saracatinib and capivasertib (1.95 mg, mole ratio = 1:1) were initially dissolved in methanol followed by adding amphiphilic polymer (20 mg in equivalent volume of chloroform). The mixture was transferred into a 10 mL round bottom flask, and the remaining procedure was performed similarly as preparation of single drug-loaded NPs. The amount of drugs loaded in the NPs was analyzed by HPLC (Agilent 1200 LC, Santa Clara, CA). The drug loading was calculated according to the calibration curve between the HPLC area values and concentrations of drug standard. The loading efficiency was defined as the ratio of drug loaded into NPs to the initial drug content. The size and size distribution of the drug-loaded NPs were measured by dynamic light scattering (DLS) instrument three times with an acquisition time of 30 s at room temperature.

### In vitro drug release testing

The drug released from the single drug-loaded NPs or co-NPs was carried out in the solution with or without CTSB. Cysteine solution in Mcllvaine’s buffer (10 mm) was added in equal volume of enzyme stock solution and pre-incubated for 5 min at 37 °C. The NPs were incubated in the buffer at 37 °C for 48 h in the presence or absence of CTSB (100 nM, pH = 5.4). A drug release control study at physiological condition (without enzyme, pH 7.4) was also performed. At predetermined time points, the samples were withdrawn and analyzed by RP-HPLC (Agilent 1200 LC, Zorbax C18 column 4.6 × 150 mm) with gradient elution.

### Animal studies and treatment regimens

All animal experiments were approved by the Institutional Animal Care and Use Committee (IACUC) of Augusta University. An equal number of male and female six-week-old NOD.Cg-*Prkdcscid Il2rgtm1Wjl/SzJ* (NSG) mice were purchased from the Jackson Laboratory (Bar Harbor, ME). An orthotopic tongue tumor model was generated as described previously [3]. Briefly, 1 × 10^5^ luciferase-containing HNSCC cells were suspended in 50 μl of PBS/Matrigel (3:1) and injected into anterior ~ 1/3 tongue of NSG mice under anesthesia. For single drug treatment, after 10 days of cell implantation, HN8- or HN12-derived tumor-bearing mice were randomized into three groups to intravenously receive vehicle (PBS), free saracatinib, or Nano-sar once every other day at 10 mg/kg body weight. For combination drug treatment, mice bearing HN12 cells were randomly assigned to one of five groups and treated intravenously once every other day with vehicle (PBS), Nano-sar, Nano-cap, the free-drug combination or Nano-com, at 10 mg/kg body weight. Mice were imaged for bioluminescent luciferase signal using a Xenogen IVIS-200 In Vivo Imaging System (PerkinElmer, Waltham, MA). When experiments were terminated, the primary tongue xenografts and major organs (including the heart, intestine, kidney, liver, lung, and spleen) from mice were excised and processed for standard histological analysis with H&E staining and immunohistochemistry (IHC).

### Determination of drug toxicity

When the animal experiment was terminated, blood was collected via ocular vein for determination of serum Alanine Transaminase (ALT/GPT), Aspartate Transaminase (AST/GOP) and creatinine. ALT and AST were measured by EnzyChromTM Alanine Transaminase Assay Kit and Aspartate Transaminase Assay kits (BioAssay System, Hayward, CA), respectively. Serum creatinine was measured by Creatinine Assay Kit (Cayman chemical, Ann Arbor, MI).

### IHC analysis

Sections of tongue tumors were immunostained with antibodies against phospho-Src, phospho-AKT, phospho-S6, and Ki67 as described previously [3, 5]. Negative controls included non-specific polyclonal rabbit antibody at 2 μg/ml (Abcam, Cambridge, MA). The sections were developed with the diaminobenzidine tetrahydrochloride (DAB) substrate kit (Vector Laboratories) and counterstained with hematoxylin.

### Apoptosis analysis

For in vitro analysis, apoptosis was determined by flow cytometry using Annexin V: PE Apoptosis Detection Kit (Invitrogen, Carlsbad, CA) with 7-AAD. For in vivo analysis, apoptosis was assessed by staining for DNA fragmentation using the DeadEnd^TM^ Fluorometric TUNEL System (Promega, Madison, WI). All TUNEL-positive cells in each section in different treatments were counted in twenty randomly selective view fields using a fluorescence microscope.

### Statistical analysis

Statistical analyses were performed using the SPSS software package version 12.0. Raw data was summarized by means, standard deviations (SD), and graphical summaries and transformed if necessary to achieve normality. Data from the in vitro experiments are presented as means ± SD from three independent experiments. Treatment effects were evaluated using one-way analysis of variance (ANOVA) at each measurement time-point. To assess the longitudinal effect of treatment, a mixed model was employed to test the overall difference across all groups as well as between each pair of groups during the whole study period. *p* < 0.05 was considered statistically significant.

## Results

### HNSCC cells exhibit differential sensitivity to saracatinib

**Fig. 1:**
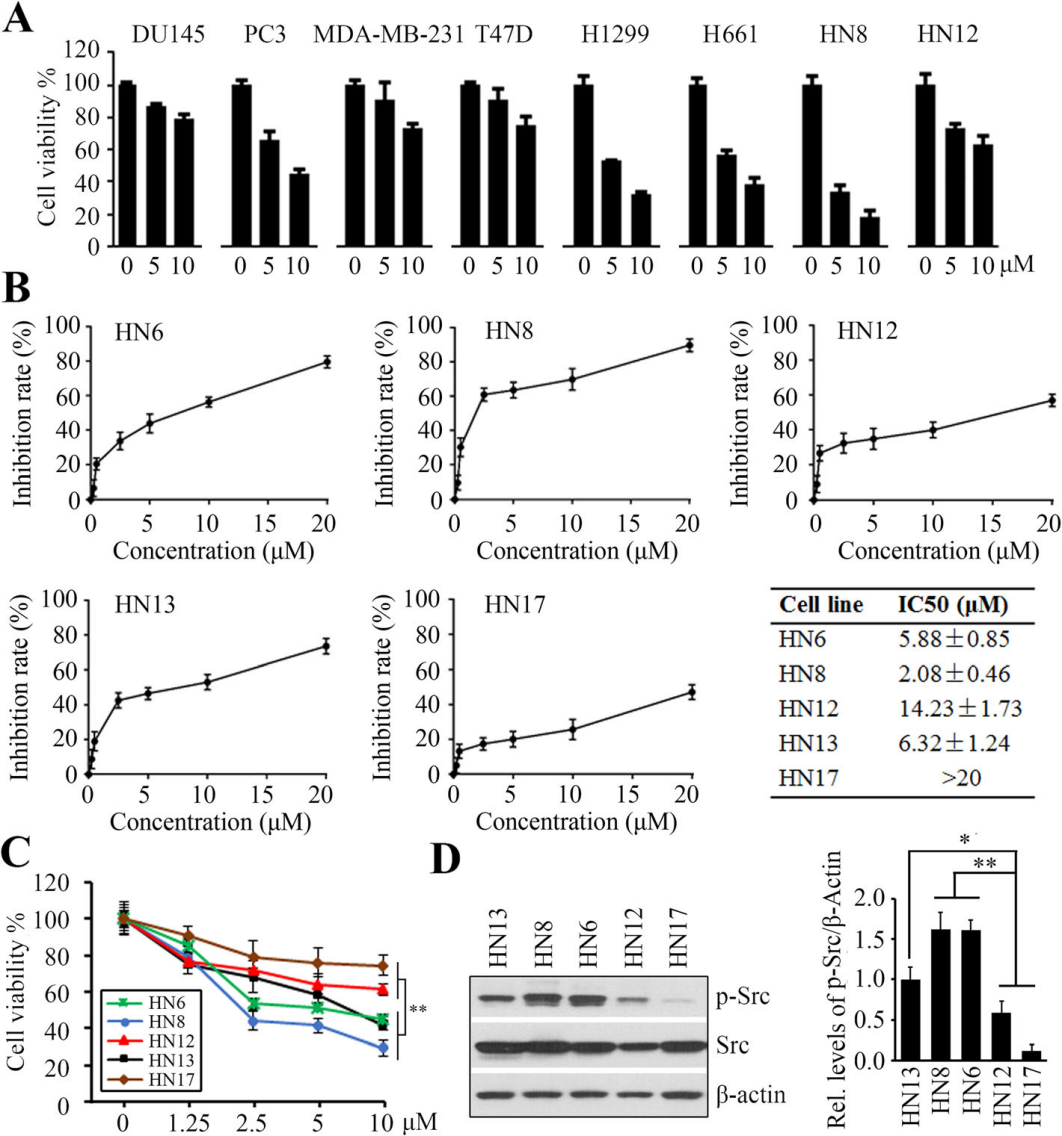
HNSCC cells exhibit differential response to saracatinib. **a** The effect of saracatinib on cell viability in different types of cancer cells determined by MTS assays on day 3 after treatment. **b** The IC50 of saracatinib in 5 different HNSCC cells determined by MTS assays on day 3 after treatment. **c** Comparison of saracatinib effect on cell viability in various HNSCC cell lines determined by MTS assays on day 3 after treatment. **d** The phosphorylation levels of Src in HNSCC cells determined by Western blotting. The representative result and quantitative data from three independent experiments were shown in the left and right panels, respectively. **p < 0.01

We first evaluated the efficacy of Src inhibitor saracatinib in several types of cancer cells. In this analysis, eight cell lines derived from prostate cancer (PC3 and DU145), breast cancer (MDA-MB-231 and T47D), lung cancer (H1299 and H1611), and HNSCC (HN8 and HN12), were treated with two doses of the Src inhibitor saracatinib for 72 h. CellTiter-Glo® Luminescent Cell Viability assay showed that saracatinib displayed cytotoxicity in all cancer cell lines examined in this study (Fig. 1a). Intriguingly, two HNSCC cell lines, HN8 and HN12, exhibited a distinct response to saracatinib (Fig. 1a). HN8 cells were more sensitive to saracatinib toxicity than other cell lines (Fig. 1a). In contrast, HN12 cells did not display a dose-dependent response to saracatinib, suggesting resistance of HN12 cells to Src inhibition (Fig. 1a). The saracatinib IC50 dose varied among HNSCC cell lines, ranging from < 7 μM in HN6, HN8 and HN13 cells, to > 14 μM in HN17 and HN12 cells (Fig. 1b). Moreover, 10 μM saracatinib induced significantly more cytotoxicity in HN6, HN8 and HN13 cells than HN17 and HN12 cells (Fig. 1c). Based upon these observations, we classified HN6, HN8, and HN13 cells as saracatinib-sensitive (Sar-S) cells (IC50 < 10 μM) and HN17 and HN12 cells as Sar-R cells (IC50 > 10 μM). These differences could not be explained by variation in the amount of Src protein; however, HN6, HN8, and HN13 cells showed significantly more Src phosphorylation than HN17 and HN12 cells (Fig. 1d), correlating with sensitivity to saracatinib (Fig. 1c, d).

### AKT inactivation enhances the anticancer activity of saracatinib in Sar-R HNSCC cells

**Fig. 2:**
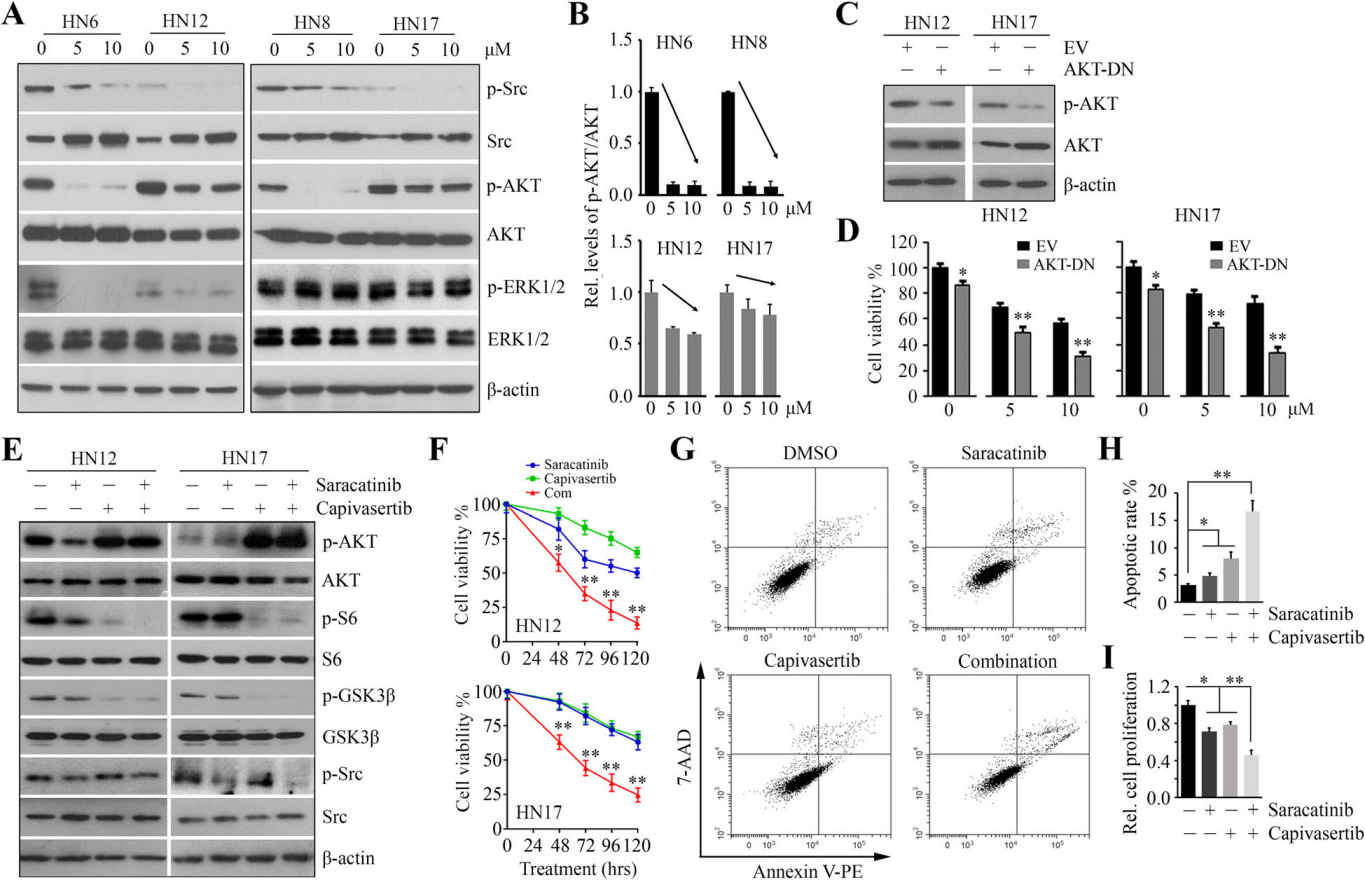
The addition of AKT inactivation augments the cytotoxicity of saracatinib in Sar-R HNSCC cells. **a** The effect of saracatinib on AKT and ERK1/2 signaling pathways in Sar-S (HN6 and HN8) and Sar-R (HN12 and HN17) HNSCC cells determined by Western blotting. **b** The quantitative data of phospho-AKT levels in saracatinib treatment from three independent experiments. **c** The effect of AKT-DN transfection on AKT activation determined by Western blotting. **d** The effect of AKT-DN transfection on cell viability in the presence or absence of saracatinib determined by CellTiter-Glo® Luminescent Cell Viability Kit on day 3 after treatment. **e** The effect of capivasertib on AKT-S6 signaling in the presence or absence of saracatinib determined by Western blotting. **f** The effect of 5 μM capivasertib on cell viability in the presence or absence of saracatinib determined by CellTiter-Glo® Luminescent Cell Viability Kit at 120 h after treatment. EV: empty vector; AKT-DN: a dominant-negative form of AKT (K179 M). **g** The effect of saracatinib, capivasertib and their combination on HN12 cell apoptosis determined on Day 3 after treatment using Annexin V: PE Apoptosis Detection Kit with 7-AAD. **h** The quantification of apoptotic rate from three independent experiments. **i** The effect of saracatinib, capivasertib and their combination on HN12 cell proliferation determined by MTS on Day 3 after treatment. *p < 0.05; **p < 0.01

We sought to understand the mechanism associated with differential saracatinib sensitivity in HNSCC cells. Both of the AKT and ERK1/2 signaling are critical for HNSCC survival and growth, and their cross-talk with Src has been identified, prompting us to determine the effects of saracatinib on these two pathways. Sar-S HN6 and HN8 cells and Sar-R HN12 and HN17 cells were treated with two doses of saracatinib for 24 h. In all four cell lines, saracatinib inhibited Src phosphorylation (Fig. 2a). Saracatinib inhibited phospho-ERK1/2 levels in HN6 and HN12 cells, but not in HN8 and HN17 cells, which did not correlate with the cytotoxicity studies (Fig. 2a). Interestingly, saracatinib markedly impaired the phosphorylation of AKT in Sar-S HN6 and HN8 cells, whereas Sar-R HN12 and HN17 cells showed no or only modest reduction in phospho-AKT (Fig. 2a, b), suggesting that the AKT pathway more relies on Src activation in HNSCC cells carrying high levels of phospho-Src. To address whether blocking AKT signaling increase the response of Sar-R HNSCC cells to saracatinib, HN12 and HN17 cells were transfected with dominant-negative AKT-DN (Fig. 2c). Expression of AKT-DN suppressed the survival of both HN12 and HN17 cells in the absence of saracatinib, as well as led to a more dramatic reduction in cell viability upon saracatinib treatment when compared with cells transfected with an empty vector (Fig. 2d). In contrast, expression of AKT-CA in Sar-S cells, such as HN8, induced a remarkable decrease in the sensitivity of saracatinib (Additional file 1: Figure S1). These data support the notion that AKT activation is tightly associated with the HNSCC response to saracatinib.

Capivasertib is a novel pyrrolopyrimidine-derived compound with great potential to inhibit AKT signaling [16, 17]. In line with a previous study [17], capivasertib induced an increase in AKT phosphorylation, but caused a decrease in AKT kinase activity, as evidenced by a decrease in the phosphorylation of AKT substrate GSK3β and S6 in both HN12 and HN17 cells (Fig. 2e). Saracatinib treatment alone slightly decreased the phosphorylation of AKT and S6 in HN12 cells and had no inhibitory effect on the AKT-S6 signaling in HN17 cells (Fig. 2e). Combined capivasertib with sacaratinib almost completely abrogated S6 phosphorylation (Fig. 2e), leading to a significant reduction in cell viability in both of these Sar-R cell lines compared with each single treatment (Fig. 2f). Consistently, additional capivasertib treatment failed to synergistically enhance saracatinib-induced lethality in Sar-S cell lines HN6 and HN8 (Additional file 2: Figure S2), which may be due to significant co-inhibition of AKT and Src by saracatinib itself in these cells.

It has been reported that EGFR activation can be regulated by the Src and/or AKT signaling in different types of cancer cells [18, 19]. We then determined the status of phospho-EGFR in saracatinib/capivasertib treatment. HN17 expressed lower levels of EGFR compared with HN12 cells, and phospho-EGFR was undetectable by Western blotting (Additional file 3: Figure S3). High levels of phospho-EGFR were detected in HN12 cells, which was inhibited by saracatinib (Additional file 3: Figure S3). Capivasertib increased the levels of phospho-EGFR, and its combination with saracatinib did not produce further suppression in phospho-EGFR compared with saracatinib treatment alone (Additional file 3: Figure S3). We also examined the status of other Src-related proteins, such as FAK and STAT3, in the presence of the indicated treatments. This analysis showed that both STAT3 and FAK, their activation was slightly or not affected in saracatinib treatment (Additional file 3: Figure S3). Although the phosphorylation levels of  FAK were dramatically inhibited in the drug combination in HN17 cells, there was no noticeable changes in phospho-FAK levels in HN12 cells (Additional file 3: Figure S3). These findings further suggest that AKT activation is one of the main mechanisms underpinning the response of HNSCC cells to saracatinib.

To further understand the drug action, we determined whether saracatinib, capivasertib, and their combination promoted apoptosis as cell viability was affected. Flow cytometry assays after Annexin V and 7-AAD staining revealed that apoptosis occurred in all indicated treatment (Fig. 2g, h). However, increase of apoptosis was much efficient in the drug combination compared with each drug alone (Fig. 2g and h). Moreover, the effect of co-treatment on suppression of cell proliferation was much greater compared with the single drug effect (Fig. 2i). Taken together, these observations strongly suggest that AKT blockade has the ability to increase the anticancer activity of saracatinib in HNSCC cells with low saracatinib sensitivity.

### AKT inactivation re-sensitizes Sar-R HNSCC cells to saracatinib

**Fig. 3:**
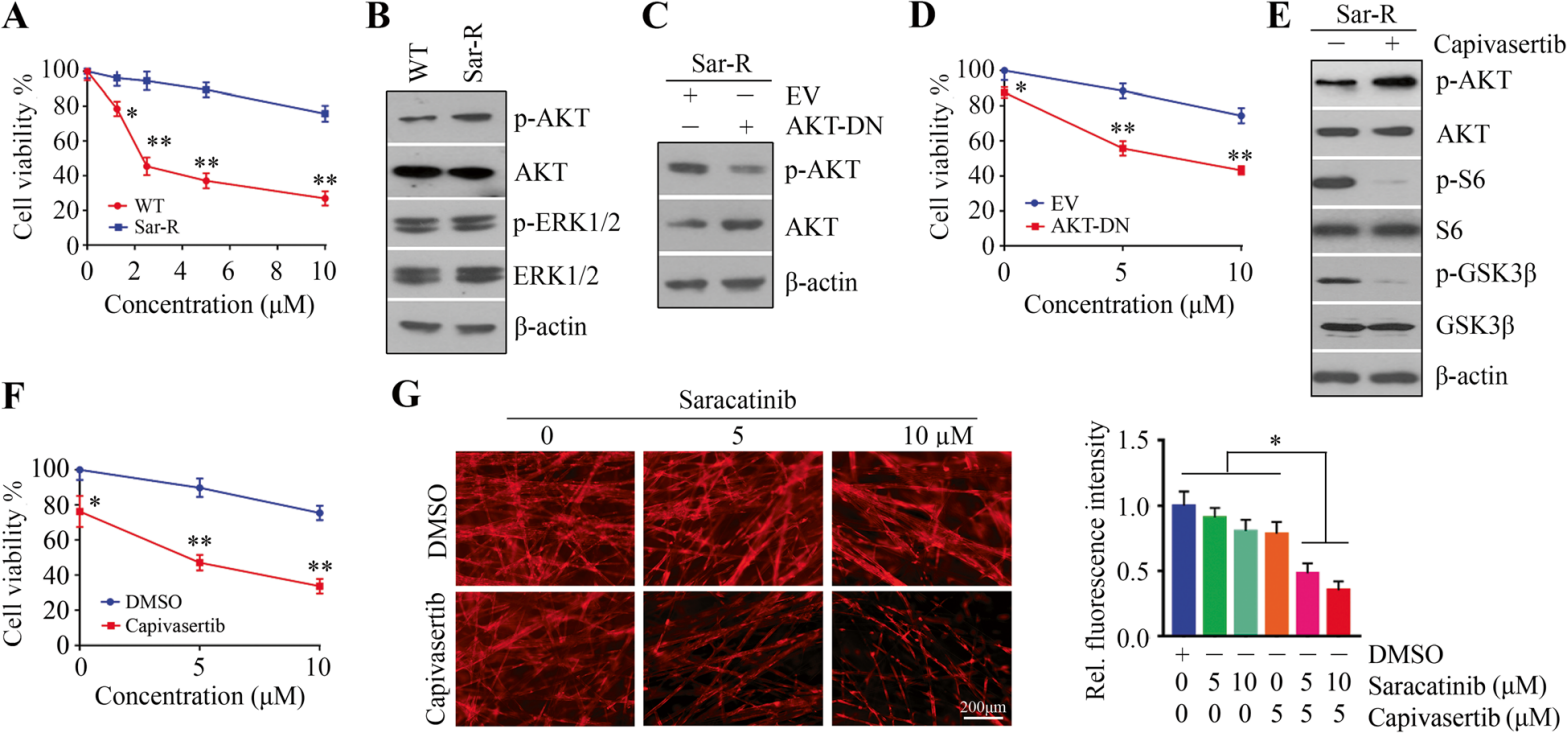
Suppressing AKT signaling resensitizes Sar-R HNSCC cells to saracatinib. **a** The effect of saracatinib on cell viability in Sar-R HN8 and the parental cells determined by CellTiter-Glo® Luminescent Cell Viability Kit on day 3 after treatment. **b** The phospho-AKT and -ERK1/2 in WT and Sar-R HN8 cells determined by Western blotting. **c** The effect of AKT-DN transfection on AKT activation in Sar-R HN8 cells determined by Western blotting. **d** The effect of AKT-DN transfection on cell viability in the presence or absence of saracatinib determined by CellTiter-Glo® Luminescent Cell Viability Kit on day 3 after treatment. **e** The effect of capivasertib on the AKT-S6 signaling in Sar-R HN8 cells determined by Western blotting. **f** The effect of capivasertib on cell viability in the presence or absence of saracatinib determined by CellTiter-Glo® Luminescent Cell Viability Kit on day 3 after treatment. **g** The effect of combined capivasertib and saracatinib on 3D cell growth on day 12 after treatment. The representative result and quantitative data from three independent experiments were shown in the left and right panels, respectively. EV: empty vector; AKT-DN: a dominant-negative form of AKT (K179 M). *p < 0.05; **p < 0.01

To further determine whether activation of AKT signaling is the possible mechanism behind saracatinib resistance in HNSCC cells, we generated Sar-R cells using Sar-S HN8 cells (Fig. 3a) as described in Materials and Methods. Compared with the parental cells, increased phospho-AKT, but not phospho-ERK1/2 was found in Sar-R cells (Fig. 3b). Phospho-AKT was attenuated by AKT-DN transfection in Sar-R cells (Fig. 3c), where cell viability was significantly decreased in the tolerated doses of saracatinib seen in cells transfected with an empty vector (Fig. 3d). We then treated Sar-R cells with 5 μM capivasertib, which induced a strong suppression on phospho-S6 (Fig. 3e). Compared with saracatinib treatment alone, saracatinib combined with capivasertib dramatically decreased the viability and 3D cell growth of Sar-R cells (Fig. 3f, g). These findings suggest that inactivation of AKT has the potential to overcome saracatinib resistance in HNSCC cells.

### Differential inactivation of AKT by saracatinib determines its therapeutic outcome for head and neck tumors in orthotopic xenograft mice

**Fig. 4:**
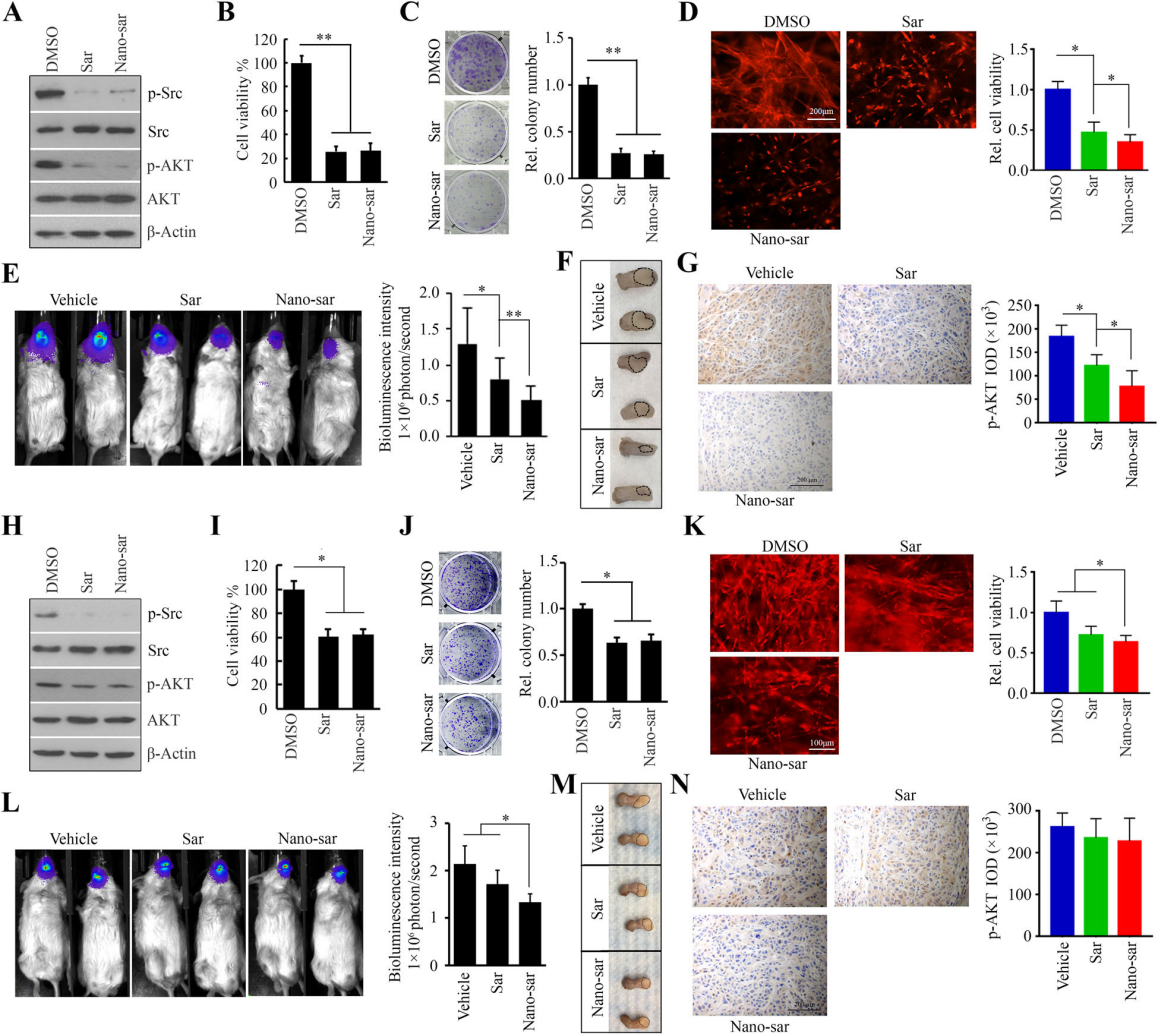
Differential inactivation of AKT by saracatinib determines its therapeutic outcome for head and neck tumors in orthotopic xenograft mice. **a**–**g** were results from HN8-derived orthotopic xenograft mice, and **h–n** were results from HN12-derived orthotopic xenograft. **a**, **h** The effect of free (Sar) and NP-associated saracatinib (Nano-sar) on the phosphorylation levels of Src and AKT. **b**, **i** The effects of free and NP-associated saracatinib on cell viability. **c**, **j** The effects of free and NP-associated saracatinib on colony formation. **d**, **k** The effects of free and NP-associated saracatinib on 3D cell growth. **e**, **l** Tumor development and progression in mice receiving the indicated treatment monitored by examining bioluminescence in the Xenogen IVIS-200 In Vivo imaging system on Day 21. The representative images and quantitative data (*n* = 5) were shown in the left and right panels, respectively. **f**, **m** The representative images showing the size of tongue tumors in mice receiving the indicated treatment. **g**, **n** The levels of phospho-AKT in orthotopic xenograft tumors receiving different treatments determined by IHC. The representative IHC images were shown in the left panel, and quantification of IHC staining using Image pro-Plus6.0 was shown in right panel. *p < 0.05; **p < 0.01

A comparison between free and NP-associated saracatinib in HN8 and HN12 cells showed that the two preparations had similarly profound effects on phospho-Src and -AKT (Fig. 4a, h) and cytotoxicity (Fig. 4b, i), without differences in 2D colony formation (Fig. 4c, j). However, Nano-sar inhibited cell growth in the 3D culture system more efficiently than free drug (Fig. 4d, k). This comparison reveals differences in drug responses between 2D and 3D cultures.

For in vivo studies, we used a more appropriate orthotopic tongue tumor model by injection of luciferase-containing cells into the anterior portion of NSG mouse tongue. Tongue xenografts established 10 days after cell inoculation, followed by intravenous treatment with vehicle, free or NP-associated saracatinib. In HN8-derived tumor-bearing mice, treatment with either free or NP-associated saracatinib resulted in significantly smaller tumor xenografts relative to those found after vehicle treatment as measured by bioluminescence, tumor size and tumor weight (Fig. 4e, f and Additional file 4: Figure S4). In line with the in vitro 3D cell growth results, tumor growth was suppressed more significantly in mice treated with Nano-sar than those treated with free saracatinib (Fig. 4e, f and Additional file 4: Figure S4). Moreover, systemic toxicity was not seen in mice receiving various treatments, as all groups maintained similar body weight (Additional file 4: Figure S4) 1 and showed no evidence of damage to major organs (Additional file 5: Figure S5). The analysis of xenografts by IHC for phospho-Src and phospho-AKT revealed the strong staining in tumors derived from vehicle-treated mice, with a high proliferative index as measured by the proportion of Ki67-positive tumor cells (Additional file 6: Figure S6). In contrast, we found less staining of phospho-Src and -AKT, as well as lower percentages of Ki67-positive cells, in tumors derived from mice treated with either free or NP-associated saracatinib (Fig. 4g and Additional file 6: Figure S6), suggesting that Nano-sar has the great potential to induce tumor shrinkage and inactivate targeting proteins than free drug. While in HN12-derived tumor-bearing mice, the xenografts showed only marginal response to saracatinib and less than 25% of reduction in the treatment with NP-associated saracatinib on Day 21 (Fig. 4l, m and Additional file 4: Figure S4). Results from IHC analysis showed that the phosphorylation of AKT did not affected by saracatinib regardless encapsulated with NPs or not (Fig. 4n). Together, our findings support the conclusion that saracatinib cannot effectively suppress head and neck tumor growth partially due to insufficiently inhibiting AKT activation in these tumor cells.

### Co-delivery NPs for saracatinib and capivasertib result in a synergistic cytotoxic effect in HNSCC cells

**Fig. 5:**
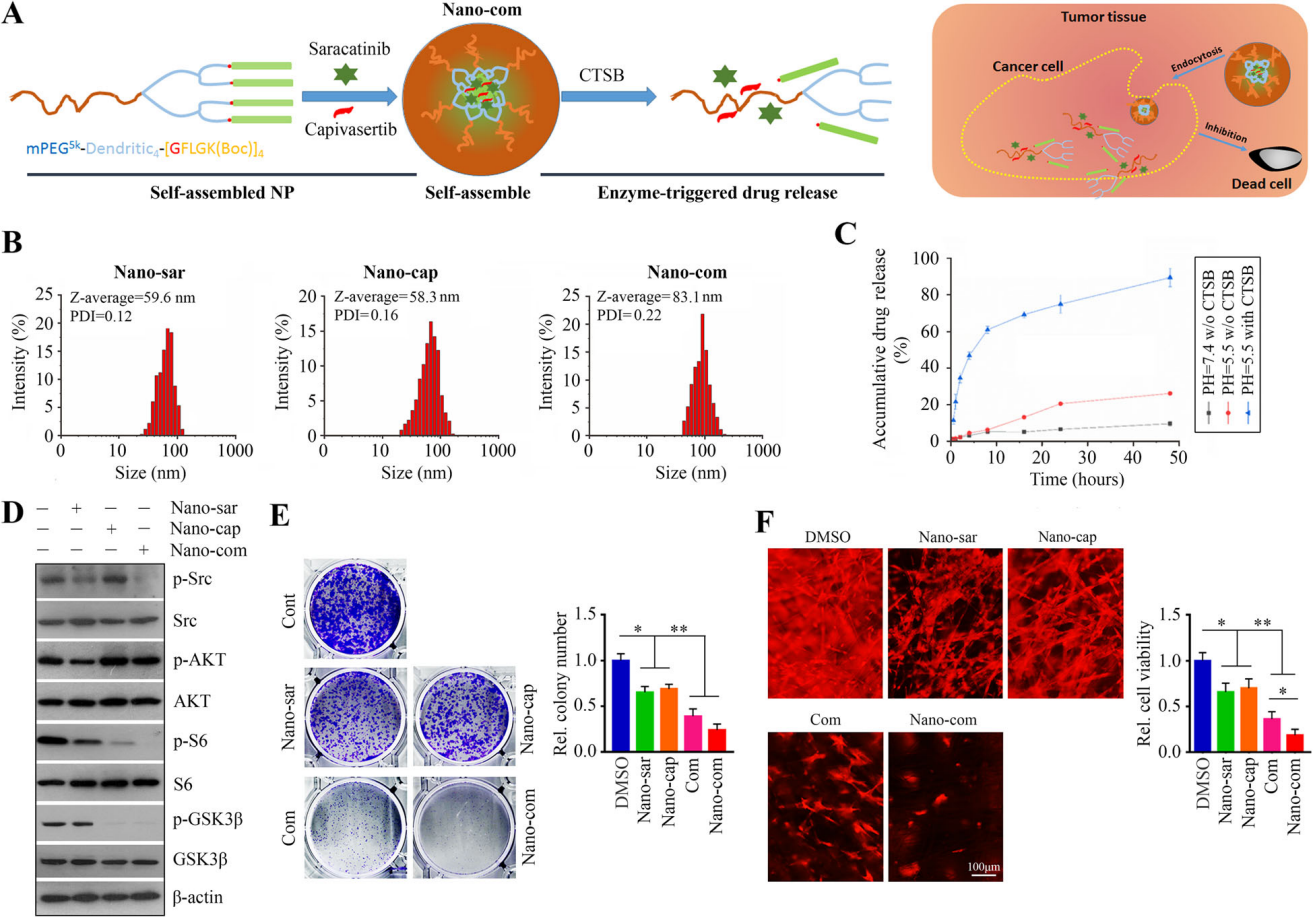
Nano-com exhibits a synergistic cytotoxic effect in HNSCC cells by co-inactivating Src and AKT signaling. **a** Schematic illustration of the self-assemble Nano-com and its disassembly upon CTSB digestion. **b** The Z-average of single and dual drug-loaded nanoscale formulations determined by DLS. **c** The drug release profile of Nano-com at various conditions determined by HPLC. **d** The effects of single and dual drug-loaded NPs on the Src and AKT activation in HN12 cells determined by Western blotting. **e** The effects of single and dual drug-loaded NPs and the free drug combination on HN12 cell colony formation on day 7 after treatment. **f** The effects of single and dual drug-loaded NPs and the free drug combination on 3D cell growth on day 12 after treatment. Nano-sar: saracitinib-loaded NPs; Nano-cap: capivasertib-loaded NPs; Com: free saracatinib/capivasertib combination; Nano-com: the saracatinib/capivasertib co-delivery NPs. *p < 0.05; **p < 0.01

The majority of therapeutic agents, including saracatinib and capivasertib, are hydrophobic in nature and are easily incorporated into NPs *via* hydrophobic interaction during the NP assembly. In this study, biocompatible and amphiphilic polymers with ideal features for drug co-delivery were used to encapsulate both saracatinib and capivasertib into NPs. A short peptide of GFLG linker was incorporated to ensure that the drugs can be released upon the lysosomal enzyme, CTSB (Fig. 5a). DLS study showed that the size of Nano-sar, capivasertib-loaded NPs (Nano-cap) and the saracatinib/capivasertib co-delivery NPs (Nano-com) were 59.6, 58.3, and 83.1 nm, respectively (Fig. 5b). As expected, Nano-com exhibited enzyme-response drug release profile, as evidenced by accelerated release of both drugs (over 80% drug release in 48 h) from NPs at pH 5.4 in the presence of CTSB (Fig. 5c). In contrast, less than 20% of the drugs was released at pH 7.4 or in the absence of CTSB (Fig. 5c), indicating that drugs encapsulated into NPs can be selectively released with the cleavage by CTSB in an acidic environment. As such, Nano-com were formulated to achieve high entrapment efficiency, small size, and controlled release behavior.

We determined the in vitro effects of single and dual drug-loaded NPs in HN12 cells. Similar to the function of free drugs, Nano-sar and Nano-cap decreased the levels of phospho-Src and phospho-S6, respectively (Fig. 5d). When HN12 cells were treated with Nano-com, both Src and S6 were inactivated (Fig. 5d), leading to improved inhibitory effects on colony formation and 3D growth as compared with single nano-drug treatments (Fig. 5e, f).

### The saracatinib/capivasertib co-delivery NPs achieve better anticancer outcomes in Sar-R head and neck tumors than monotherapy in mice

**Fig. 6:**
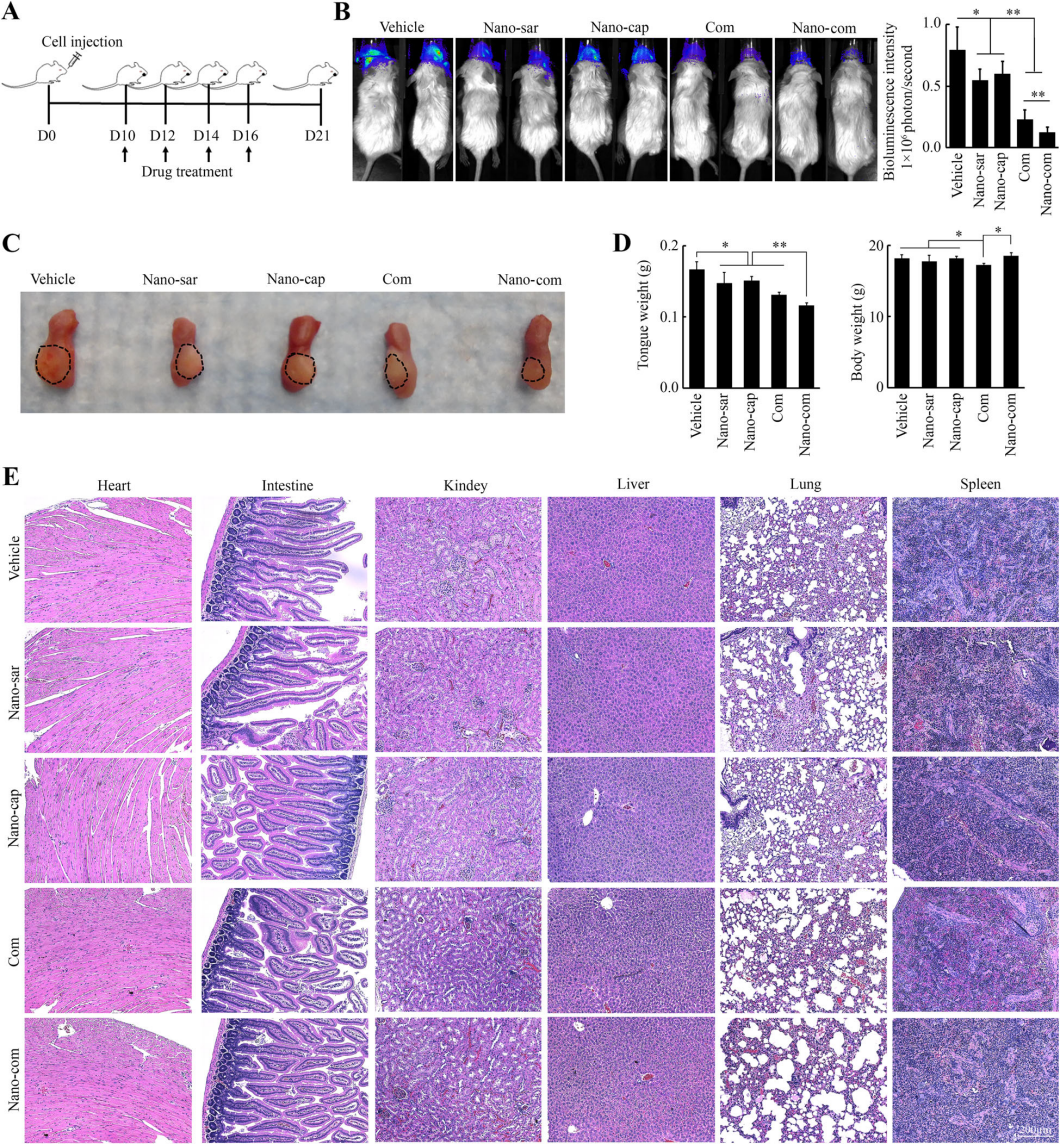
Nano-com achieves a superior effect on Sar-R head and neck tumor growth than monotherapy in orthotopic xenograft mice. **a** The flowchart of drug treatment process. **b** Tumor development and progression in mice receiving different treatments monitored by examining bioluminescence in the Xenogen IVIS-200 In Vivo imaging system on Day 21. The representative images and quantitative data (*n* = 5) were shown in the left and right panels, respectively. **c** The representative images showing the size of tongue tumors in mice receiving the indicated treatment. **d** The average weight of tongue and body in different treatment groups. **e** Histology examination of tissues taken from mouse major organs (heart, intestine, kidney, liver, lung and spleen) at the endpoint of each treatment. Nano-sar: saracitinib-loaded NPs; Nano-cap: capivasertib-loaded NPs; Com: free saracatinib/capivasertib combination; Nano-com: the saracatinib/capivasertib co-delivery NPs. *p < 0.05; **p < 0.01

These exciting in vitro data encouraged us to evaluate the therapeutic efficacy of Nano-com in mice. In in vivo study, HN12-derived tumor-bearing NSG mice received vehicle, Nano-sar, Nano-cap, free or NP-associated drug combination every other days for four times (Fig. 6a). After treatment, tumor burden was reduced in each single-arm treatment as evidenced by lower bioluminescent signal (Fig. 6b), smaller tumor size (Fig. 6c), and decreased tumor weight (Fig. 6d). Combination of saracatinib with capivasertib achieved superior anticancer effects than either treatment alone (Fig. 6b–d). Most importantly, the reduction in tumor growth was more significant in mice receiving Nano-com than those receiving free drug combinations (Fig. 6b–d). Our data show that at least in a preclinical model, the nano-based co-delivery system that incorporates saracatinib and capivasertib is a very efficient and effective method to target Sar-R HNSCC.

A rational drug interaction should increase the benefit of the administered drugs without increasing side effects. To test the hepatotoxicity and nephrotoxicity profiles of NP-associated drugs, we measured serum ALT, AST and creatinine. AST, ALT and creatinine levels were significantly elevated in mice receiving free drug combination (Additional file 7: Figure S7), suggesting that combination of saracatinib with capivasertib would induce systemic toxic. In contrast, biochemical tests revealed no liver or kidney toxicity in the NP-associated combined drug treatment group (Additional file 7: Figure S7). Moreover, H&E staining showed no morphologic changes in the major organs (Fig. 6e). These observations indicate that nano drugs we developed do not produce detectable systemic toxicities.

**Fig. 7:**
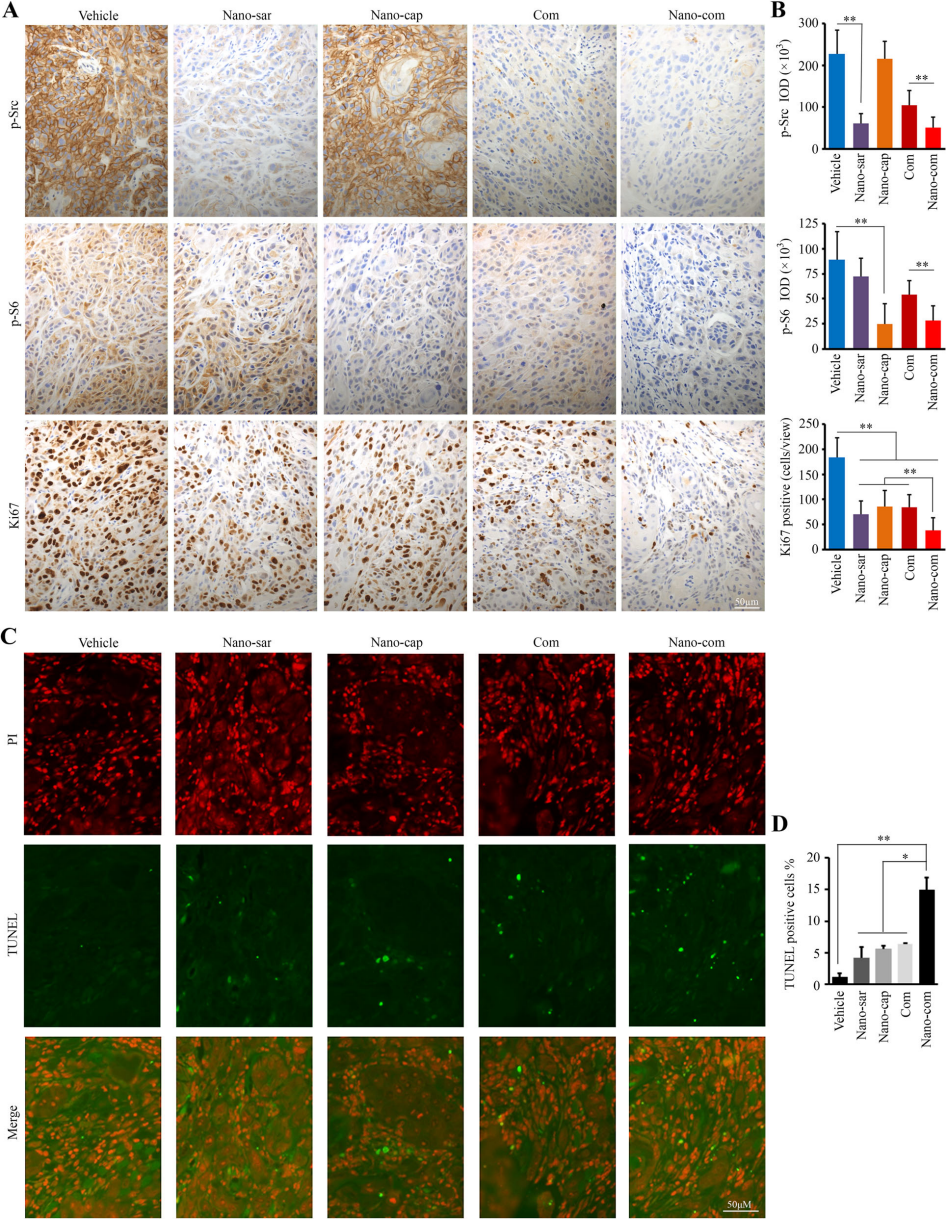
Nano-com improves the anti-HNSCC effect by co-inhibiting Src and AKT signaling in orthotopic xenograft mice. **a**, **b** The levels of phospho-Src, phospho-S6 and Ki67 in orthotopic xenograft tumors receiving different treatments determined by IHC. The representative IHC images were shown in (**a**) and quantification of IHC staining using Image pro-Plus6.0 was shown in (**b**). **c**, **d** The levels of apoptosis in orthotopic xenograft tumors receiving different treatments determined by TUNEL assays. The representative TUNEL-stained xenograft sections were shown in (**c**) and quantification of TUNEL-positive tumor cells was shown in (**d**). Nano-sar: saracitinib-loaded NPs; Nano-cap: capivasertib-loaded NPs; Com: free saracatinib/capivasertib combination; Nano-com: the saracatinib/capivasertib co-delivery NPs. *p < 0.05; **p < 0.01

To further evaluate the effects of treatments on the tumor xenografts, sections were immunostained with the antibodies against phospho-Src, phospho-S6 and Ki67. Consistent with the in vitro data (Fig. 5d), Nano-sar decreased phospho-Src, but had no effect on S6 activation, while Nano-cap treatment significantly abrogated S6 phosphorylation without affecting Src activation (Fig. 7a, b). Both phospho-Src and phospho-S6 levels were significantly reduced in xenografts from mice treated with Nano-com when compared with monotherapy (Fig. 7a, b). Moreover, Nano-com suppressed Src and S6 activation in xenografts more than did the free-drug combination (Fig. 7a, b). Moreover, TUNEL assays revealed that Nano-com was more robust in the induction of tumor cell apoptosis than either single-arm treatment or non-NP based drug combination, as evidenced by the observation of the highest number of TUNEL-positive cells in the group with Nano-com treatment (Fig. 7c, d). Collectively, our study provides evidence that in a preclinical orthotopic model, co-delivery of capivasertib and saracatinib by tumor-targeting NPs is safe and highly effective.

## Discussion

HNSCC exhibiting resistance to single molecular-targeted therapeutics poses a challenge to its effective clinical management, and novel augmentation/combination strategies are actively sought to improve treatment outcomes. We and others have reported the constitutive activation of Src in HNSCC and implicated that high Src activation on treatment selection for HNSCC [5, 20, 21]. In the present study, we further show that high levels of Src phosphorylation correlate with high sensitivity of HNSCC cells to saracatinib, and whether saracatinib results in a sufficient AKT inactivation is strictly associated with its sensitivity in HNSCC cells. Our study also shows diverging results in HNSCC cells that saracatinib can potently abrogate the phosphorylation levels of AKT in Sar-S cells, but it can only marginally affect AKT activation in Sar-R cells. It seems that the AKT signaling is more dependent on Src activation in HNSCC cells carrying high levels of phospho-Src compared with those carrying low levels of phospho-Src. Additional suppression of AKT activation in saracatinib treatment can increase efficacy of Src inhibition in HNSCC patients with activated AKT and even overcome saracatinib resistance. The findings reported here provide a strong rationale for combined Src and AKT inhibitors against HNSCC harboring these activation.

Saracatinib has been explored to give a sustained blockage of AKT in lung cancer cells, with the effect comparable with the PI3K-AKT inhibitor LY294002 [22]. Interestingly, we found that LY294002 was 3~5 times less potent than capivasertib on S6 inactivation in HNSCC cells, which may explain why LY294002 at the doses less than 10 μM fails to produce a synergistic effect in combination with saracatinib, though it can induce stronger cytotoxicity in HNSCC cells compared with capivasertib at the same dose. It appears that the S6 signaling contributes specifically to saracatinib resistance in HNSCC with activated AKT, where S6 phosphorylation depends on AKT activation rather than Src activation. As such, it enhances the ability of HNSCC cells to redirect growth promotion to the AKT-S6 salvage survival pathway in Src inhibition. Recently, one published clinical study shows that patients with PIK3CA-mutated breast or gynecologic cancer were given the capivasertib from 480 to 800 mg in the clinic [17]. The maximum plasma concentration (C_max_) was around 2500 ng/ml (equal to 5.8 μM) in 640-mg-dose group, suggesting that 5 μM we used in this study should be the pharmacologically achievable concentration of capivasertib in humans. Therefore, the evidence offered here provides the rational basis for the use of capivasertib to induce suppression of AKT in saracatinib treatment, and suggests that saracatinib warrants further study in combination with S6 inhibitors.

Because low efficacy and drug resistance are commonly associated with the use of a single agent for cancer treatment, combination therapy remains a particularly attractive approach to overcome these clinical challenges. One main aim of the drugs used in combination is to achieve synergistic therapeutic effect. To determine the broad acceptance in scientific applications, the additive, synergistic, or antagonistic nature of the combined drug effect must be examined. Synergism is more than an additive effect and antagonism is less than an additive effect [23]. For the combination of capivasertib and saracatinib, drug combination index (CI) was calculated at different dose combinations before the therapeutic evaluation. The resulting CI was much less than 1 (0.38 in HN12 cells and 0.47 in HN17 cells) when HNSCC cells were co-treated with capivasertib and saracatinib at 5 μM each drugs for 3 days, suggesting that additional AKT blockade by capivasertib to saracatinib has the potential to produce a strong synergistic effect.

Src family kinases can mediate both epidermal growth factor receptor (EGFR)-dependent and -independent pathways, and even serve as upstream activators of EGFR [21, 24]. EGFR is considered as a prime target for new therapy against HNSCC as its overexpression correlates with advanced disease stage and poor prognosis in HNSCC patients [25, 26]. Currently, none of EGFR-targeting has shown benefit over the standard of care. It has been explored that addition of Src inhibitor to EGFR inhibitor erlotinib in HNSCC cells can enhance the overall anti-growth effects of either drug alone [27]. It looks that saracatinib has the potential to inhibit EGFR activation in some HNSCC cells, such as HN12 cells. However, this effect may be cellular context-dependent. The interaction of saracatinib with the EGFR signaling still needs to be determined in a large range of HNSCC cell lines and clinical samples. In our study, we also compared the anti-HNSCC effects of saracatinib combined with capivasertib to its combination with erlotinib. The result showed that the CI of the combination of saracatinib and erlotinib was higher than that of the combination of saracatinib and capivasertib at the same dose of each drugs (0.51 *vs* 0.38 in HN12 cells, and 0.58 *vs* 0.47 in HN17 cells), suggesting that additional capivasertib treatment is worthy of future clinical investigation in HNSCC patients who are lack of clinical efficacy of anti-Src single agent.

Multiple drugs delivered systemically can enter both the tumor and critical organs (e.g., the heart and lung), and overlapping of toxicity is common as one of the obstacles for the development of a successful drug combination. Although co-treatment with capivasertib and saracatinib induced significant tumor repression, increased toxicity was observed in mice due to the nonselective nature of these drugs in their free form. To address combination therapy-induced systemic toxicity, a feasible strategy is to encapsulate the nonselective therapeutic drugs into NPs that can carry the drugs specifically into the tumor. Recently, many smart NP systems with higher targeting specificity on tumor have been reported [28–30]. Because profound upregulation of CTSB was found in HNSCC tissues compared with paired adjacent normal tissues [5, 31], CTSB-sensitive NPs were developed to achieve tumor-targeting capacity. The dual drug-loaded NPs developed in the present study optimize the properties and pharmacokinetics of combined drugs and release them into the same population of tumor cells. Moreover, these NPs exhibit strong synergistic anti-HNSCC effects with no additive or synergistic toxicity in major organs, providing an efficient way for improving survival for HNSCC patients.

## Conclusions

In summary, we unveil that the insufficient suppression of the AKT-S6 signaling axis is one of the mechanisms associated with decrease clinical benefits of Src-targeted treatment in HNSCC patients, and co-inhibition of Src and AKT represents a compelling strategy for achieving superior anti-HNSCC effects in those patients harboring these activations. We also discover advances in the co-delivery of Src and AKT inhibitors using tumor-targeting NPs in order to enhance the efficacy of cancer treatment, which has potentially translational impact on anticancer drug design and execution.

## Supplementary information

**Additional file 1: Figure S1.** Constitutive activation of AKT signaling enhances the resistance of saracatinib in HN8 cells. (A) The effect of AKT-CA transfection on AKT activation determined by Western blotting. (B) The effect of AKT-CA transfection on cell viability in the presence or absence of saracatinib determined by CellTiter-Glo® Luminescent Cell Viability Kit on day 3 after treatment. *p<0.05; **p<0.01.

**Additional file 2: Figure S2.** The effect of indicated treatment on cell viability determined by CellTiter-Glo® Luminescent Cell Viability Kit at 72 hours after treatment. *ns*: not significant; *p<0.05; **p<0.01.

**Additional file 3: Figure S3.** The effect of indicated treatment on other Src-related proteins (including STAT3, FAK and EGFR) in HNSCC cells.

**Additional file 4: Figure S4.** The average weight of tongue and body in HN8- (A) and HN12-derived orthotopic xenograft mice (B) during different treatments. *p<0.05; **p<0.01.

**Additional file 5: Figure S5.** Histology examination of tissues taken from mouse major organs (heart, intestine, kidney, liver, lung and spleen) at the endpoint of each indicated treatment.

**Additional file 6: Figure S6.** The levels of phospho-Src and Ki67 in HN8-derived orthotopic xenograft tumors receiving different treatments determined by IHC. The representative IHC images were shown in (A) and quantification of IHC staining using Image pro-Plus6.0 was shown in (B). *p<0.05; **p<0.01.

**Additional file 7: Figure S7.** Blood biochemical indexes of NSG mice following intravenous administration of indicated treatment. In this study, AST (A) and ALT (B) levels reflect hepatic functions, and creatinine (C) levels reflect nephron functions. *p < 0.05; **p < 0.01.

## Data Availability

All data generated or analyzed during this study are included in the manuscript and its supplementary information files.

## Abbreviations

3D: Three-dimensional
ALT: Alanine transaminase
AST: Aspartate transaminase
CML: Chronic myeloid leukemia
CTSB: Cathepsin B
DLS: Dynamic light scattering
EGFR: Epidermal growth factor receptor
HNSCC: Head and neck squamous cell carcinomas
HPLC: High-performance liquid chromatography
IHC: immunohistochemistry
NP: Nanoparticle
NSG: NOD.Cg*-Prkdc*^*scid*^ *Il2rg*^*tm1Wjl*^*/SzJ*
*S*ar-S: Saracatinib-sensitive
Sar-R: Saracatinib-resistant
