# Supplementary material for: Simultaneously inactivating Src and AKT by saracatinib/capivasertib co-delivery nanoparticles to improve the efficacy of anti-Src therapy in head and neck squamous cell carcinoma
Source: J Hematol Oncol. 2019 Dec 5;12:132. doi: 10.1186/s13045-019-0827-1 (PMC6896687; doi:10.1186/s13045-019-0827-1)
Supplement: Supplementary file 1 — Additional file 1: Figure S1. Constitutive activation of AKT signaling enhances the resistance of saracatinib in HN8 cells. (A) The effect of AKT-CA transfection on AKT activation determined by Western blotting. (B) The effect of AKT-CA transfection on cell viability in the presence or absence of saracatinib determined by CellTiter-Glo® Luminescent Cell Viability Kit on day 3 after treatment. *p<0.05; **p<0.01. [file 13045_2019_827_MOESM1_ESM.docx]

**
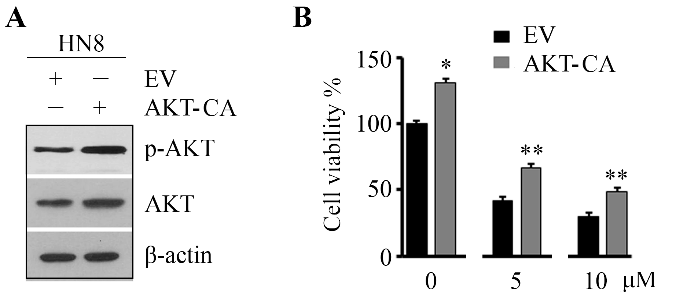
**

**Figure S1: Constitutive activation of AKT signaling enhances the resistance of saracatinib in HN8 cells.** (**A**) The effect of AKT-CA transfection on AKT activation determined by Western blotting. (**B**) The effect of AKT-CA transfection on cell viability in the presence or absence of saracatinib determined by CellTiter-Glo^®^ Luminescent Cell Viability Kit on day 3 after treatment. **p*<0.05; ***p*<0.01.
