# Supplementary material for: Simultaneously inactivating Src and AKT by saracatinib/capivasertib co-delivery nanoparticles to improve the efficacy of anti-Src therapy in head and neck squamous cell carcinoma
Source: J Hematol Oncol. 2019 Dec 5;12:132. doi: 10.1186/s13045-019-0827-1 (PMC6896687; doi:10.1186/s13045-019-0827-1)
Supplement: Supplementary file 2 — Additional file 2: Figure S2. The effect of indicated treatment on cell viability determined by CellTiter-Glo® Luminescent Cell Viability Kit at 72 hours after treatment. ns: not significant; *p<0.05; **p<0.01. [file 13045_2019_827_MOESM2_ESM.docx]

**
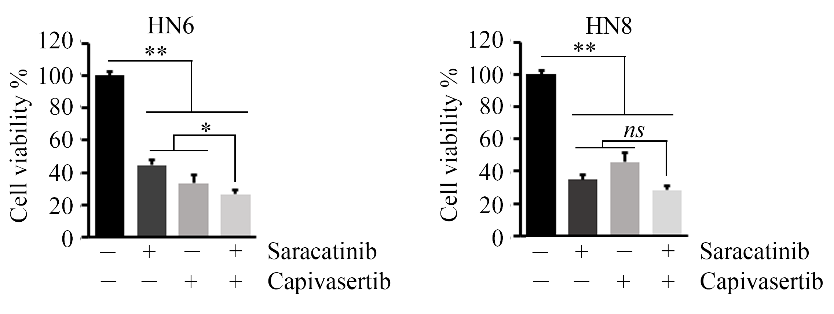
**

**Figure S2:** The effect of indicated treatment on cell viability determined by CellTiter-Glo^®^ Luminescent Cell Viability Kit at 72 hours after treatment. *ns*: not significant; **p*<0.05; ***p*<0.01.
