# Supplementary material for: Simultaneously inactivating Src and AKT by saracatinib/capivasertib co-delivery nanoparticles to improve the efficacy of anti-Src therapy in head and neck squamous cell carcinoma
Source: J Hematol Oncol. 2019 Dec 5;12:132. doi: 10.1186/s13045-019-0827-1 (PMC6896687; doi:10.1186/s13045-019-0827-1)
Supplement: Supplementary file 3 — Additional file 3: Figure S3. The effect of indicated treatment on other Src-related proteins (including STAT3, FAK and EGFR) in HNSCC cells. [file 13045_2019_827_MOESM3_ESM.docx]

**
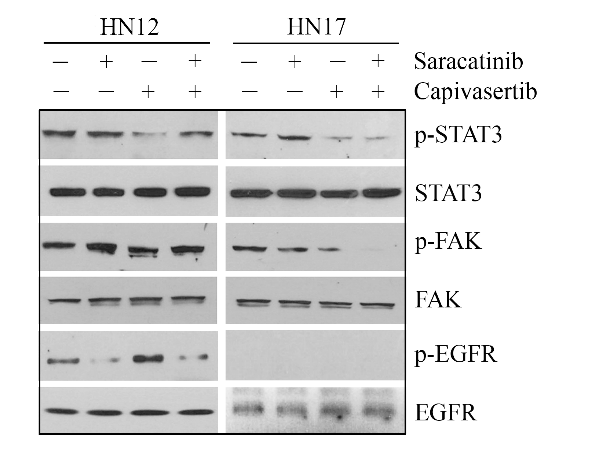
**

**Figure S3:** The effect of indicated treatment on other Src-related proteins (including STAT3, FAK and EGFR) in HNSCC cells.
