# Supplementary material for: Simultaneously inactivating Src and AKT by saracatinib/capivasertib co-delivery nanoparticles to improve the efficacy of anti-Src therapy in head and neck squamous cell carcinoma
Source: J Hematol Oncol. 2019 Dec 5;12:132. doi: 10.1186/s13045-019-0827-1 (PMC6896687; doi:10.1186/s13045-019-0827-1)
Supplement: Supplementary file 4 — Additional file 4: Figure S4. The average weight of tongue and body in HN8- (A) and HN12-derived orthotopic xenograft mice (B) during different treatments. *p<0.05; **p<0.01. [file 13045_2019_827_MOESM4_ESM.docx]

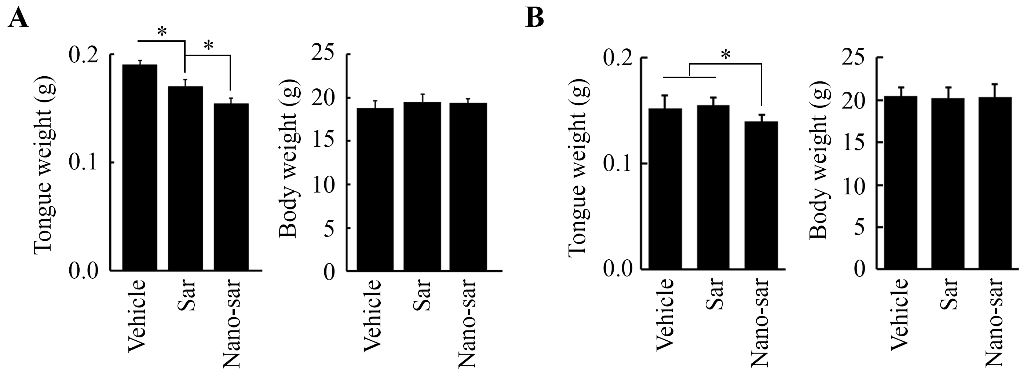


**Figure S4:** The average weight of tongue and body in HN8- (**A**) and HN12-derived orthotopic xenograft mice (**B**) during different treatments. **p*<0.05; ***p*<0.01.
