# Supplementary material for: Simultaneously inactivating Src and AKT by saracatinib/capivasertib co-delivery nanoparticles to improve the efficacy of anti-Src therapy in head and neck squamous cell carcinoma
Source: J Hematol Oncol. 2019 Dec 5;12:132. doi: 10.1186/s13045-019-0827-1 (PMC6896687; doi:10.1186/s13045-019-0827-1)
Supplement: Supplementary file 5 — Additional file 5: Figure S5. Histology examination of tissues taken from mouse major organs (heart, intestine, kidney, liver, lung and spleen) at the endpoint of each indicated treatment. [file 13045_2019_827_MOESM5_ESM.docx]

**
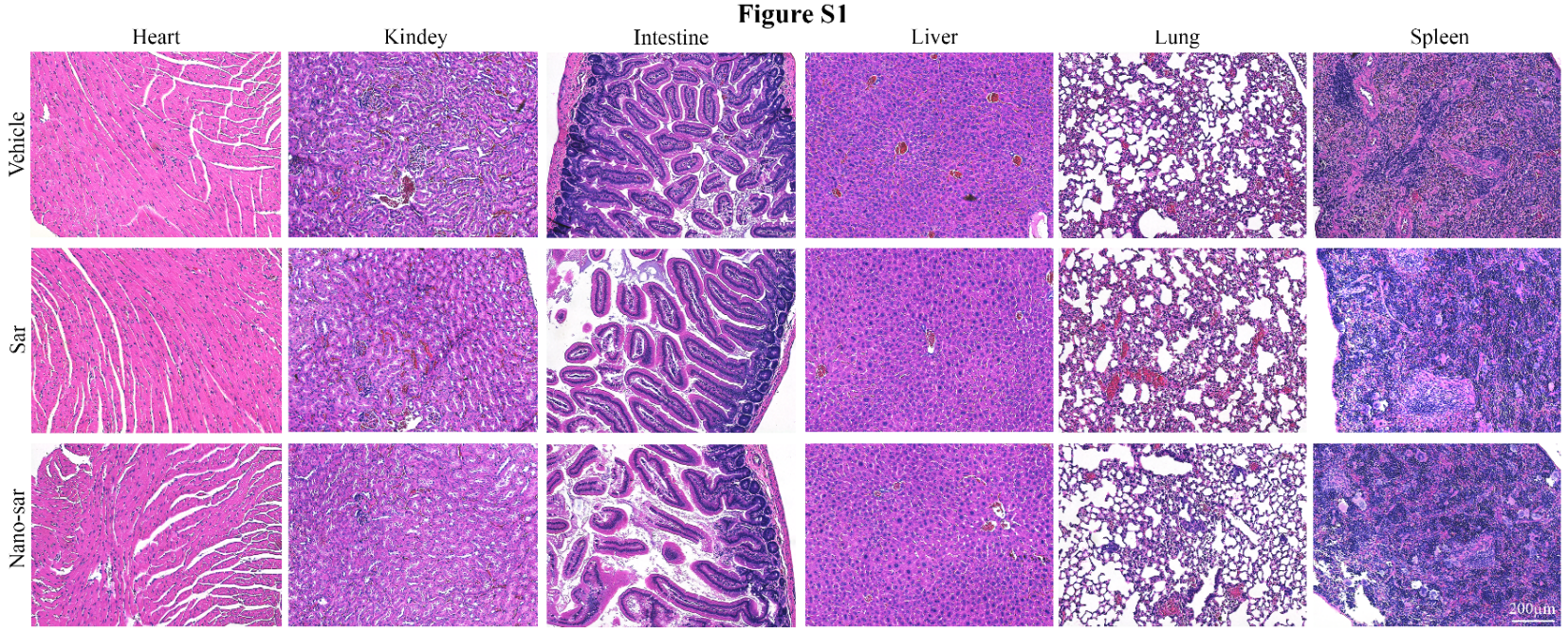
**

**Figure S5:** Histology examination of tissues taken from mouse major organs (heart, intestine, kidney, liver, lung and spleen) at the endpoint of each indicated treatment.
