# Supplementary material for: Simultaneously inactivating Src and AKT by saracatinib/capivasertib co-delivery nanoparticles to improve the efficacy of anti-Src therapy in head and neck squamous cell carcinoma
Source: J Hematol Oncol. 2019 Dec 5;12:132. doi: 10.1186/s13045-019-0827-1 (PMC6896687; doi:10.1186/s13045-019-0827-1)
Supplement: Supplementary file 6 — Additional file 6: Figure S6. The levels of phospho-Src and Ki67 in HN8-derived orthotopic xenograft tumors receiving different treatments determined by IHC. The representative IHC images were shown in (A) and quantification of IHC staining using Image pro-Plus6.0 was shown in (B). *p<0.05; **p<0.01. [file 13045_2019_827_MOESM6_ESM.docx]

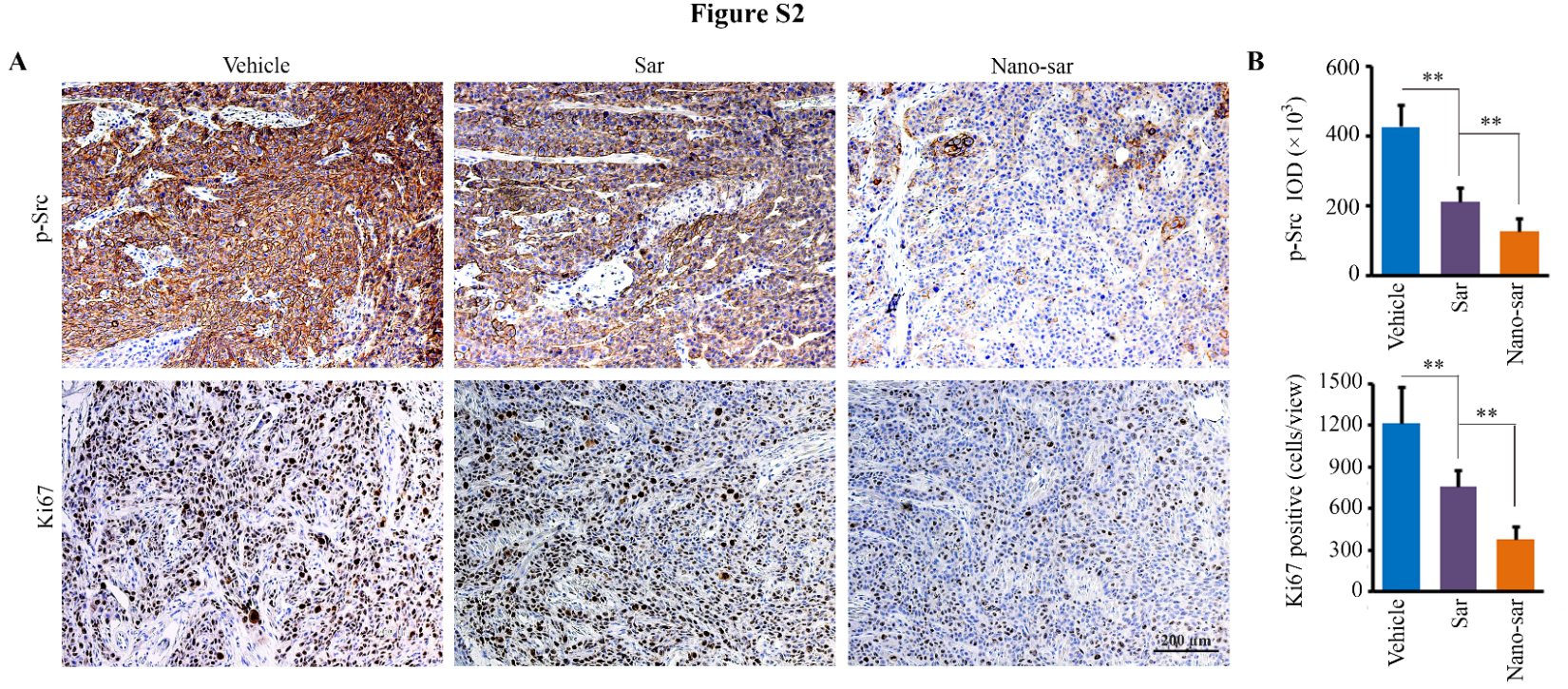
 **Figure S6:** The levels of phospho-Src and Ki67 in HN8-derived orthotopic xenograft tumors receiving different treatments determined by IHC. The representative IHC images were shown in (A) and quantification of IHC staining using Image pro-Plus6.0 was shown in (B). **p*<0.05; ***p*<0.01.
