# Supplementary material for: Simultaneously inactivating Src and AKT by saracatinib/capivasertib co-delivery nanoparticles to improve the efficacy of anti-Src therapy in head and neck squamous cell carcinoma
Source: J Hematol Oncol. 2019 Dec 5;12:132. doi: 10.1186/s13045-019-0827-1 (PMC6896687; doi:10.1186/s13045-019-0827-1)
Supplement: Supplementary file 7 — Additional file 7: Figure S7. Blood biochemical indexes of NSG mice following intravenous administration of indicated treatment. In this study, AST (A) and ALT (B) levels reflect hepatic functions, and creatinine (C) levels reflect nephron functions. *p < 0.05; **p < 0.01. [file 13045_2019_827_MOESM7_ESM.docx]

**Figure S7:** Blood biochemical indexes of NSG mice following intravenous administration of indicated treatment. In this study, AST (A) and ALT (B) levels reflect hepatic functions, and creatinine (C) levels reflect nephron functions. **p* < 0.05; ***p* < 0.01.
